# Abdominal and pancreatic motion correlation using 4D CT, 4D transponders, and a gating belt

**DOI:** 10.1120/jacmp.v14i3.4060

**Published:** 2013-05-06

**Authors:** Ricardo Betancourt, Wei Zou, John P. Plastaras, James M. Metz, Boon‐Keng Teo, Alireza Kassaee

**Affiliations:** ^1^ Radiation Oncology University of Pennsylvania Medical Center Philadelphia PA USA

**Keywords:** pancreatic motion, respiratory motion, 4D electromagnetic transducer system, gating belt system, image‐guided radiation therapy, beam gating radiotherapy, tracking

## Abstract

The correlation between the pancreatic and external abdominal motion due to respiration was investigated on two patients. These studies utilized four dimensional computer tomography (4D CT), a four dimensional (4D) electromagnetic transponder system, and a gating belt system. One 4D CT study was performed during simulation to quantify the pancreatic motion using computer tomography images at eight breathing phases. The motion under free breathing and breath‐hold were analyzed for the 4D electromagnetic transponder system and the gating belt system during treatment. A linear curve was fitted for all data sets and correlation factors were evaluated between the 4D electromagnetic transponder system and the gating belt system data. The 4D CT study demonstrated a modest correlation between the external marker and the pancreatic motion with R‐square values larger than 0.8 for the inferior–superior (inf‐sup). Then, the relative pressure from the belt gating system correlated well with the 4D electromagnetic transponder system's motion in the anterior–posterior (ant‐post) and the inf–post directions. These directions have a correlation value of −0.93 and 0.76, while the lateral only had a 0.03 correlation coefficient. Based on our limited study, external surrogates can be used as predictors of the pancreatic motion in the inf–sup and the ant–post directions. Although there is a low correlation on the lateral direction, its motion is significantly shorter. In conclusion, an appropriate treatment delivery can be used for pancreatic cancer when an internal tracking system, such as the 4D electromagnetic transponder system, is unavailable.

PACS number: 87.55.kh

## I. INTRODUCTION

Pancreatic cancer is one of the most deadly cancers, with a 1% five‐year overall survival.^1^, ^2^ At the time of diagnosis, around 60% of the patients have locally advanced disease and/or distant metastasis.^3^, ^4^, ^5^ Even when patients have metastatic disease, a significant percentage will die of local complications, according to a John Hopkins Rapid Autopsy Program in Pancreatic Cancer.

External beam radiotherapy (EBRT) may be delivered as a radical or palliative treatment for pancreatic cancer. In order for radiotherapy to be successful, the entire tumor volume must be irradiated to high doses. However, this can pose some challenges due to tumor motion and radiation tolerance of organs at risk. As treatment delivery techniques such as intensity‐modulated radiation therapy (IMRT), volumetric‐modulated arc therapy (VMAT), stereotactic body radiation therapy (SBRT) advance, knowledge of target position become more crucial.^6^, ^7^, ^8^, ^9^, ^10^ For example, tumor motion may result in a different dose distribution if motion is not correctly accounted for. Several techniques have been utilized to improve the localization and precise delivery of the treatment. Langen and Jones^(11)^ and others^12^, ^13^, ^14^, ^15^, ^16^, ^17^ have reviewed techniques to manage interfraction and intrafraction motion. In particular, Langen and Jones reviewed the literature for pancreatic motion and concluded that the pancreas can move up to 3.0 cm during normal breathing and up to 8.0 cm in deep breathing. Covering such motion with wide margins would be inappropriate, since a large volume of healthy organs, such as bowel and kidneys, might receive therapeutic doses.^(6)^ Hence, it is important to understand how the pancreas moves.

Ozhasoglu and Murphy^(18)^ have described several approaches to optimize treatment delivery when the target is affected by breathing motion. One approach is to use breath‐hold or to modify the breath cycle.^19^, ^20^, ^21^, ^22^, ^23^ Other methods include synchronizing the radiation beam on time to the breathing cycle to deliver the dose when the target moved the least,^24^, ^25^, ^26^, ^27^, ^28^ and allowing the patient to breathe normally and track the tumor position during treatment.^29^, ^30^, ^31^ Also, the motion of an external marker is often used as a reference to deduce the internal motion of the tumor.^32^, ^33^, ^34^ Consequently, evaluating the correlation between an external marker on the surface of the body and the pancreatic motion is crucial for understanding and evaluating treatment delivery. Previous studies have investigated the correlation between abdominal motion and external markers using 2D techniques.^31^, ^35^, ^36^, ^37^, ^38^ This study aims to investigate the correlation between the external abdominal motion and the internal pancreatic tumor motion. First, the correlation was found between the position of an external marker placed at the abdomen region and three transducers inside the pancreatic tumor using 4D CT. Then, another correlation between the information given by a gating belt system and location of three 4D electromagnetic transponders was evaluated.

## II. MATERIALS AND METHODS

For this study, two patients (A and B) with pancreatic cancer were enrolled on an IRB‐approved prospective protocol. Electromagnetic transponders were surgically implanted around the patient's pancreatic tumor, as previously described.^39^, ^40^, ^41^ Only one 4D CT was acquired for each patient. Each 4D CT was reconstructed to eight 3D CTs according to different phases. The spatial resolution was set to 0.1 cm (lateral), 0.1 cm (ant–post) and 0.3 cm (inf–sup) directions. The patients were treated using a 3D conformal technique planned using the Eclipse version 8.6 (Varian Medical Systems, Palo Alto, CA). Patients were monitored with the gating belt system and the 4D electromagnetic transponder system for 20 fractions and for at least 5 minutes in each fraction, 2.5 minutes before treatment and 2.5 minutes during treatment.

### A. Correlation using 4D CT

The Real‐time Position Management (RPM) Respiratory Gating System (Varian Medical Systems) was utilized for the 4D CT studies. In the RPM system, a block has two circular reflective markers which reflect infrared light emitted from a set of infrared sources mounted in a ring around the camera lens. This infrared light is detected by the camera lens and is sent to the RPM respiratory gating system. Using this information, a 4D CT study was performed on each patient yielding a CT image dataset in eight phases. The location of one of the circular reflective markers was recorded and compared to the 3D center location of the three 4D electromagnetic transducers acquired from the CT datasets. A linear curve was fitted in each direction and its R‐squared values were calculated.

### B. Correlation using a 4D electromagnetic transponder system and a gating belt system

The gating belt system uses mechanical pressure on a pressure‐sensitive device imbedded in the belt to detect the external respiratory motion (pressure change) in real time.^(12)^ The 4D electromagnetic transponder system, on the other hand, yields the 3D coordinates of each individual transducer and the center of the three‐transducer ensemble.^42^, ^43^, ^44^ The implanted transducers in patients are easily localized using a 4D electromagnetic array, as described by Balter.^(13)^ Briefly, the 4D electromagnetic array excites the transducers and receives their signal. The location of the receiver with respect to the room is acquired by infrared sensors located in the room. At the time of treatment, patients were positioned using the 4D electromagnetic transducer localization and tracking system. The gating belt was placed on the umbilical area of each patient. Both the relative gating belt system motion due to the respiratory cycle and the internal motion of the transducers were recorded using the software for each system. Data were acquired for around 2.5 minutes before treatment. Then, both patients were asked to take three big breaths and hold it voluntarily for each field delivery. The breath holds were done at inhale and with patient's comfort. Each breath hold lasted for about 30 sec. Using the information yielded by these systems, the correlation between the relative amplitude from the gating belt system and the 3D motion of the 4D electromagnetic transducers was analyzed. Each analysis was done on free‐breathing and breath‐hold datasets.

## III. RESULTS

### A. Correlation using 4D CT

**Figure 1:**
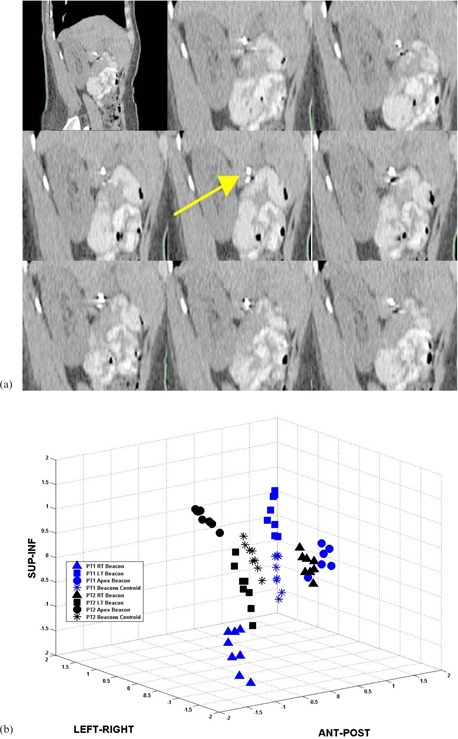
4D CT sagittal images (a) for Patient A illustrating the right transducer location through eight phases of breathing cycle. 3D plot (b) illustrating the location of the three transducers for Patient A and Patient B. The triangles, squares, circles, and asterisks represent the right, left, apex, and center of mass transducers, respectively.

**Figure 2:**
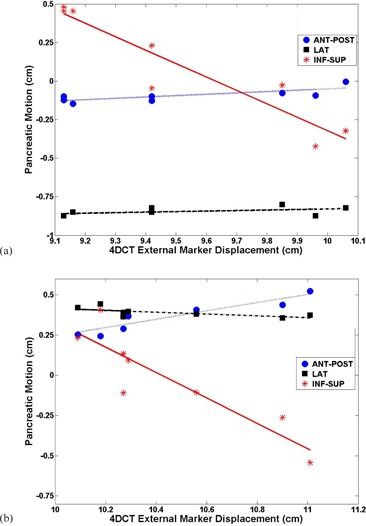
Pancreatic positions in three dimensions as a function of ant–post external marker position from 4D CT studies for Patient A (a) and Patient B (b). The linear least squares fits for the points are also illustrated for each direction.

The 4D CT studies for both patients indicated that breathing affects pancreatic motion mainly in two directions, inf–sup and ant–post. These results compared favorably to those from Mori et al.^(45)^ Figure 1(a) illustrates eight different phases of the breathing cycle for Patient A. In this figure, the right transponder, marked by a yellow arrow, moved mainly in the inf–sup direction, with a smaller displacement along the ant–post direction. Figure 1(b) illustrates the location of all three transponders for both patients during a breathing cycle, as well as the center of mass of the transponders characterized by asterisks. In this case, the maximum displacements were 0.90 cm, 0.14 cm, and 0.07 cm in the inf–sup, ant–post, and lateral directions for Patient A, respectively. For Patient B, they were 0.95 cm, 0.28 cm, and 0.09 cm, respectively. The external marker's displacement was 0.93 cm and 0.92 cm for Patient A and for Patient B, respectively. Figure 2 illustrates absolute position of the 4D electromagnetic transponders for eight phases of the breathing cycle. Its position was given as a function of the external marker position from 4D CT studies for both patients, namely Patient A (left) and Patient B (right). For Patient A, the peak‐to‐peak amplitudes were 0.15 cm, 0.07 cm, and 0.90 cm in the ant–post, lateral, and inf–sup directions, respectively. The linear fits had R‐squared values of 0.60 (ant‐post), 0.23 (lateral), and 0.88 (inf–sup). The lowest R‐square value was in the lateral direction. However, the movement was the lowest in this axis, with amplitude of 0.07 cm. The highest R‐square values resulted from the inf–sup direction, which had the highest range of ± 0.90 cm according to 4D CT. Patient B demonstrated similar results. The peak‐to‐peak amplitudes varied from 0.28 cm, 0.08 cm, and 0.95 cm in the ant–post, lateral, and inf–sup directions, respectively. The corresponding linear fits had R‐square values of 0.84 (A–P), 0.45 (lateral), and 0.81 (I–S).

### B. Correlation using a 4D electromagnetic transducer system and a gating belt system: free breathing

**Figure 3:**
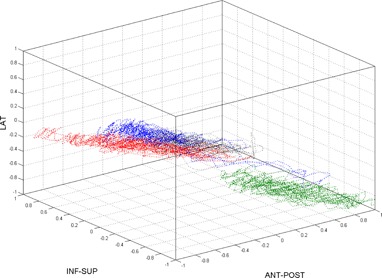
3D pancreatic motion for Patient A as give by the 4D electromagnetic transducer system for four sessions. Each image illustrates the 3D motion of the center of mass of the three transducers for 120 sec in free‐breathing motion.

Figure 3 illustrates the 4D electromagnetic transducer motion for Patient A during 120 sec for four sessions. Clearly, the pancreatic motion was not cyclic and appeared to be irregular. Although the displacement was concentrated within a small region, the center of motion changed from day to day, as seen from Fig. 3 where the center of motion was concentrated at different locations. The significance of the motion path location within the cube is that the 4D electromagnetic transducers have a cyclic motion but also an unpredictable displacement.

**Figure 4:**
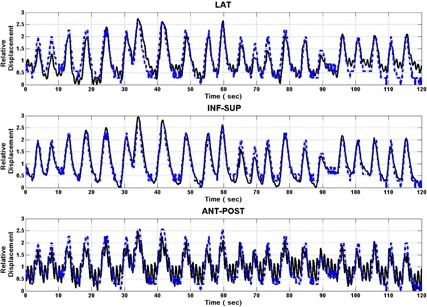
For Patient A, the pancreatic lateral (top), inf–sup (middle), and ant–post (bottom) motions for 120 sec as given by 4D electromagnetic transducer system (solid line), and the abdominal ant–post motion as given by gating belt system (broken line). The curves were plotted using their relative magnitudes to better appreciate their correlation.

**Figure 5:**
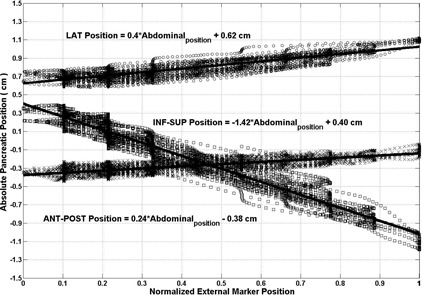
Pancreatic positions in 3D given by 4D electromagnetic transducer system vs. external abdominal ant–post position given by gating belt system during free‐breathing motion. A linear curve was fitted in each direction: lateral (circle), inf–sup (square), and ant–post (asterisk).

In Fig. 4, one case of the relationship between the 4D transponder system and gating belt system motions is illustrated for Patient A. In this figure, the 4D electromagnetic transducer in lateral (top), inf–sup (middle), and ant–post locations for 120 sec (solid line) and the abdominal pressure as given by the gating belt system (dashed line) were plotted as a function of time. The plots were normalized to the mean value better appreciate their correlation. Clearly, the ant–post and the inf–sup motion of the 4D electromagnetic transducer completely overlapped with gating belt pressure. On the other hand, the lateral motion of the 4D electromagnetic transducer illustrated here was one of the ones that best correlated with the abdominal pressure. All other cases demonstrated a lower correlation. In Fig. 5, a linear fit between these motions are illustrated. This figure illustrates the free‐breathing motion. In this case, the gating belt system data were normalized to unity since it was not calibrated to yield an exact displacement. In this example, all 4D electromagnetic transponder system data points had a linear relationship with the normalized gating belt system points. For instance, the lateral movement had a slope value of 0.4 with a Y‐intercept of 0.62 cm. This linear relationship translates to a pancreatic position at 0.82 cm, with a total translational motion of ± 0.20 cm in the coordinate system defined during the CT simulation. Even though this motion was small, the pancreas had an even smaller displacement range, with a given location of −0.26 cm with a range of ± 0.12 cm for the ant–post direction. However, the largest displacement was along the inf–sup direction with a slope of −1.42 cm and a Y‐intercept of 0.40 cm. In this case, the longitudinal location of the pancreas can be given by −0.31 cm with a large range of ± 0.71 cm. The negative sign indicated a 180° phase shift between the internal motion and the gating belt system data. During the course of the treatment, 20 sessions were observed and evaluated. The slope was calculated for each direction: lateral, inf–sup, and ant–post. The mean slope value and its standard deviation were (0.03 cm, 0.21 cm), (‐1.12 cm, 0.13 cm), and (0.33 cm, 0.12 cm) for the lateral, inf–sup, and ant–post directions, respectively. The lowest standard deviations were those for the inf–sup and ant–post directions that were about one half of the standard deviation for the lateral direction. Also, the correlation coefficient was highest for the inf–sup direction, with an average of −0.93. This direction also had the lowest standard deviation of 0.04. The ant–post motion also had a relatively modest correlation coefficient of 0.76, with a standard deviation of 0.09. Finally, the lateral motion had the lowest correlation of the three directions, ranging from 0.87 to −0.70, with a mean value of 0.03 and a standard deviation of 0.42. This indicates that the motion in the lateral direction is poorly correlated with the external surrogate during respiration.

### C. Correlation between 4D electromagnetic transducer system and gating belt system: multiple breath holds

**Figure 6:**
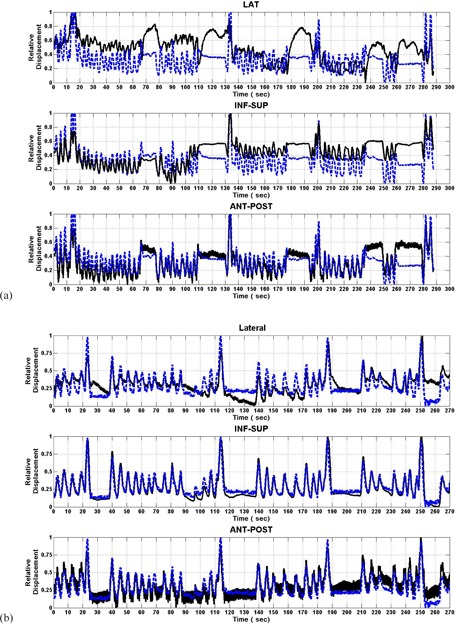
Pancreatic motion for Patient A (a) and Patient B (b) for a 270 and 290 sec period, respectively. These were taken with intermittent breath‐hold periods. The pancreatic lateral (top), inf–sup (middle), and ant–post (bottom) motions as given by Calypso (solid line), and the abdominal ant–post motion as given by the gating belt system (broken line). The curves were plotted using their relative magnitudes.

**Figure 7:**
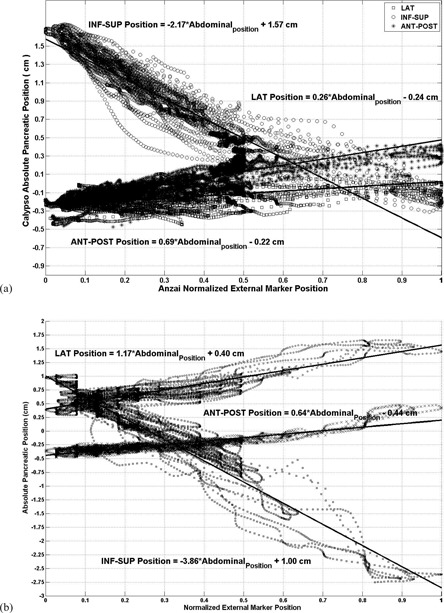
Pancreatic position in 3D given by Calypso vs. external abdominal ant–post position given by the gating belt system during an intermittent breath‐hold motion for Patient A (a) and Patient B (b). The gating belt system data were normalized to unity. A linear curve was fitted in each direction: lateral (square), inf–sup (circle), and ant–post (asterisk).

Figure 6 illustrates the motion of the 4D electromagnetic transducer for Patient A (right) and Patient B (left) for a 270 and 290 sec periods, respectively. These measurements were taken with intermittent breath‐hold periods to evaluate the effect of breath holding. The transducer's lateral (top), inf–sup (middle), and ant–post motions are given by the solid line, and the relative pressure of the gating belt system by the dashed line. Patient A clearly demonstrated a strong correlation between the 4D electromagnetic transponder system and the gating belt system data, as illustrated by the overlapping structures. However, the 4D electromagnetic transponder system and the gating belt system disagreed between the relative magnitudes during breath hold, especially in the lateral dimension where the 4D electromagnetic transponder system yielded a lower magnitude than gating belt system. In Patient B, the 4D electromagnetic transponder system yielded a higher relative magnitude than gating belt system. Figure 7 illustrates a linear fit between the 4D electromagnetic transponder system and the gating belt system for both patients with intermittent breath‐hold motion. As seen in the free‐breathing motion, all 4D electromagnetic transponder system data points also have a linear relationship with the normalized gating belt system points. A key difference from the free‐breathing motion data was that there was a major displacement increase along all directions, especially in the sup–inf motion. For example, the longitudinal motion had a slope value of −3.9 cm with a Y‐intercept of 1.0 cm. This linear relationship translated to a pancreatic position at −2.9 cm with a range of ± 1.9 cm. Transitioning between breath hold and breathing (catching breath) could have caused a change in breathing pattern that affected the linear fit differently in each session.

Although the transducer's motion also increased in the other two dimensions, its displacement was lower than 1.0 cm. Another noteworthy characteristic in these plots was that the 4D electromagnetic transponder system's points for gating belt system values of less than ∼0.5 follow the linear pattern for the fitting curve quite tightly, whereas the 4D electromagnetic transponder system points for gating belt system values greater than ∼0.5 showed a wider spread.

The slopes of the linear fitted curves for all sessions for Patient A and for Patient B were also calculated for each direction (lateral, inf–sup, and ant–post). For Patient A, the mean slope value and its standard deviation are (0.3 cm, 0.52 cm), (‐2.66 cm, 0.72 cm), and (0.48 cm, 0.22 cm) for the lateral, inf–sup, and ant–post directions, respectively. In this case, ant–post yielded the lowest standard deviation, with the inf–sup direction having the highest of all three. Patient B had similar results; the mean slope value and its standard deviation were (0.05 cm, 0.23 cm), (‐2.08 cm, 0.54 cm), and (0.64 cm, 0.12 cm) for the lateral, inf–sup, and ant–post directions, respectively. The correlation coefficients were the highest for the inf–sup motion, with an average of −0.95 for Patient A and −0.78 for Patient B. Although for Patient A this motion still had the lowest spread as demonstrated by the lowest standard deviation of 0.09, it had a larger spread for Patient B with a standard deviation of 0.14. Similarly, the ant–post and the lateral motions yielded a lower correlation coefficient of 0.65 and 0.20 for Patient A, and 0.72 and 0.11 for Patient B, respectively, with larger standard deviation values in all four cases.

## IV. DISCUSSION

Many techniques have been used to minimize the effect of motion on dose delivery such as image‐guided radiation therapy, beam gating, breath hold or any combination of these. However, an important parameter for the utilization of these techniques is the target motion during treatment. Frequently, large margins are utilized to guarantee exposing the target to therapeutic radiation doses. However, increasing the margins increases toxicity. Therefore, it is important to understand and possibly predict how the target is moving. In this study, pancreatic motion was evaluated using two methods, namely a 4D CT study and a 4D electromagnetic system combined with a gating belt system.

The first method involved 4D CT in which an external marker and eight CT images reconstructed at different breathing cycles were used. Inherently, this technique provided a low temporal resolution tool to investigate pancreatic motion and is prone to 4D CT imaging artifacts related to breathing irregularities. Furthermore, although the 4D CT images were reconstructed in specific breathing cycles, these images only provided the average displacement of the pancreas at specific respiratory phases, and the pancreas does not follow a periodic displacement. In fact, the pancreas had a very irregular movement. Also, the use of 4D CT only gives information at a single time point and there may be motion changes throughout the treatment course. Consequently, 4D electromagnetic transducer system was an essential tool to characterize the pancreatic motion with a high temporal resolution of about 0.1 sec.

The second method to correlate pancreatic with abdominal motions involved using the 4D electromagnetic transducer system and the gating belt system, both of which have higher spatial and temporal resolution. The 4D electromagnetic transducer allowed for full characterization of the pancreatic motion. From these data, it was confirmed that displacement is not cyclic and its center was displaced from day to day. With the 4D electromagnetic transducer system and gating belt system, it was possible to fully correlate the external abdominal and pancreatic motions. This was true especially for free‐breathing motion. Due to this correlation, it was possible to estimate the pancreatic motion given the external abdominal motion by a linear curve.

When breath‐hold techniques were utilized for this study, it was noticed that the proportionality constants for all three dimensions increased, especially for the inf–sup direction, that went from −1.1 cm to −2.7 cm — a total increase of 137%. This agreed with the assertion by Langen and colleagues^(11)^ that pancreatic motion can increase from 3.0 cm during normal breathing to up to 8.0 cm in deep breathing. While the correlation factors showed a minor increase from −0.93 to −0.95, the standard deviation of the slopes increased from 0.13 cm to 0.72 cm. The reason for an increased pancreatic displacement during breath holding was that patients inhaled or exhaled allowing the pancreas to move longer distances for short periods of time. Furthermore, the inhale/exhale is not reproducible.

In a treatment, patient was instructed for three types of breathing: a free breathing, a deep inhalation before breath hold, and a breath hold after inhalation while breathing normally. For patient treated with either type of breath hold, the residual motion during breath hold, the reproducibility of motion between different breath holds, and the correlation of internal–external motion within breath hold are most important. In this study, it was noticed that for most cases, the residual motion during the breath hold was within 0.10 cm for the lateral motion, within 0.20 cm for the ant–post motion, and within 0.30 cm for the inf–sup motion. However, the reproducibility of the motion between different breath holds was less certain. For example, within the same treatment day, the 4D electromagnetic transducers often displayed different motions, namely on one breath hold the transducer could be slightly moving upward by 0.30 cm while in the subsequent two breath holds, they stayed within 0.05 cm. Finally, since the position of the 4D transducers varied between breath holds, their correlation was small.

## V. CONCLUSIONS

In this investigation, a pilot study was undertaken to investigate the correlation of external marker with pancreatic motion using 4D CT, 4D electromagnetic transducer system, and a gating belt system. 4D CT yielded a very strong correlation between the three 4D electromagnetic transducers and the circular reflective marker in spite of its low temporal resolution. On the other hand, based on the very limited number of patients, there appears to be a reasonable correlation between the pancreatic movement (the three 4D electromagnetic transducers) and the external surrogate (pressure belt in this case). This means that the pancreatic treatments can be gated based on the surrogate motion for places when the use of internal markers is not feasible.
